# Syringe micro vibrator (SMV) a new device being introduced in dentistry to alleviate pain and anxiety of intraoral injections, and a comparative study with a similar device

**DOI:** 10.1186/1750-1164-5-1

**Published:** 2011-01-07

**Authors:** Amir Hashem Shahidi Bonjar

**Affiliations:** 1 Student of Dentistry, Students Research Committee, School of Dentistry, Shahid Beheshti Medical University, Evin, Tehran 1983963113, Iran

## Abstract

**Background:**

Neurologically, it is proven that stimulation of larger diameter fibers - e.g. using appropriate coldness, warmth, rubbing, pressure or vibration- can close the neural "gate" so that the central perception of itch and pain is reduced. This fact is based upon "Gate-control" theory of Melzack and Wall.

**Presentation of the hypothesis:**

Syringe Micro Vibrator is a new design being introduced for the first time in the field of Dentistry. This device is a promising breakthrough in pain and anxiety management and may deliver solution for clinicians plagued with patient pain phobia. It has an off-set rotating micro vibration creator with ultra high frequency and ultra low altitude that can be easily placed on any standard dental syringe and some disposable syringes. This device was registered as an invention in dentistry and received Iran National Patent number of 63765.

**Testing the hypothesis:**

By creating micro vibration, this device would be effective in reducing the pain and anxiety confronted with most types of intraoral injections as palatal, mandibular block, intraligamental and local infiltration. From the aspect of the patient pain management, this device contributes both physiologically (based on Gate Control Theory of pain) and psychologically (based on the device function as will be explained by dentist to the patient as a modern pain reducing technology). From the aspect of clinician, SMV motor provides vibrations with ultra high frequency to alleviate pain, but since it has ultra low vibration altitude, it has no adverse effect on the clinician dexterity and accuracy during injection and it does not interfere with pin point localization of injection site.

**Implications of the hypothesis:**

Upon mounting on a conventional dental anesthesia injection syringe, SMV is switched on and the clinician then uses normal injection technique to administer the anesthetic. This device is not only a useful accessory device for ordinary patients, but also more useful for pediatric patients and those who have a phobia of intraoral injection or pain.

## Background

Pain is an unpleasant sensory and emotional experience associated with actual or potential tissue damage, or described in terms of such damage [1]. Pain management, also called algiatry, employs an interdisciplinary approach for easing the suffering and improving the quality of life of those experiencing pain [2]. Because of the fear of pain in dental injections, some people avoid, cancel or do not appear for dental appointments [3]. Pain and anxiety control is one of the most important aspects in administration of local anesthetic in dental practice. Administration of local anesthetic produces pain and anxiety that may cause subsequent unfavorable behavior [4]. The levels of, and relationships between dental fear, general fears and phobias were studied by Berggren in 109 adult patients at a specialized dental fear clinic using two dental fear scales [5]. The results indicated that a large proportion of these dentally fearful individuals were prone to injection fear. The pain induced by infiltration of local anesthetic agents can be reduced in a number of complementary methods which include application of topical analgesics such as methocaine [6], suggestion [7], distraction techniques [8], counter irritation [9,10], varying the rates of infiltration [11], buffering the local anesthesia [12-15], reduced speed of injection [16,17] and use of vibration [18-23]. Melzack and Schecter [24] showed that itch can be reduced by vibration of stimulated area. They concluded that their results may be attributed to physiological activities occurring at the early stages of information transmission. Vibratory stimulation is a potential method for the treatment of pain. It is one of several non-pharmacological techniques used to reduce pain [25]. The effects of vibration on pain have been reported in both clinical [21,26] and experimental settings [18,27]. Vibration activates both superficial and deeply located receptors [18,22,28]. Lundeberg *et al *proved reduction of pain during vibratory stimulation in patients suffering acute or chronic musculoskeletal pain of different origin. They noticed that sixty-nine per cent of the patients reported a reduction of pain during vibratory stimulation [19,20,27]. Nanitsos *et al *investigated the effect of vibration on pain during local anesthesia injections. Their results indicated that compared to no vibration-stimulus injections, injections with vibration resulted in less pain and lower pain rating by studied patients [19]. Based on gate control theory [29,30], mechanisms of pain relief induced by vibration are elaborated by several workers suggesting pain can be reduced by simultaneous activation of nerve fibers that conduct non-noxious stimuli [1,19,20]. In this regard Longe *et al *[31] and Aminabadi [9] indicated that counter stimulation reduces pain perception. They concluded that when vibration is applied as a counter stimulation to an anesthetic injection, it will reach the brain before the pain sensation does. The brain can perceive only one sensation at a time; therefore, the sensation that arrives at the brain first is the one that will be felt. Syringe Micro Vibrator is a new design being introduced for the first time in the field of Dentistry. As a vibration stimulus, structural constituents, analogy and the role of SMV to alleviate patient pain and anxiety during dental anesthesia injection would be discussed hereafter.

## Presentation of the hypothesis

**Figure 1:**
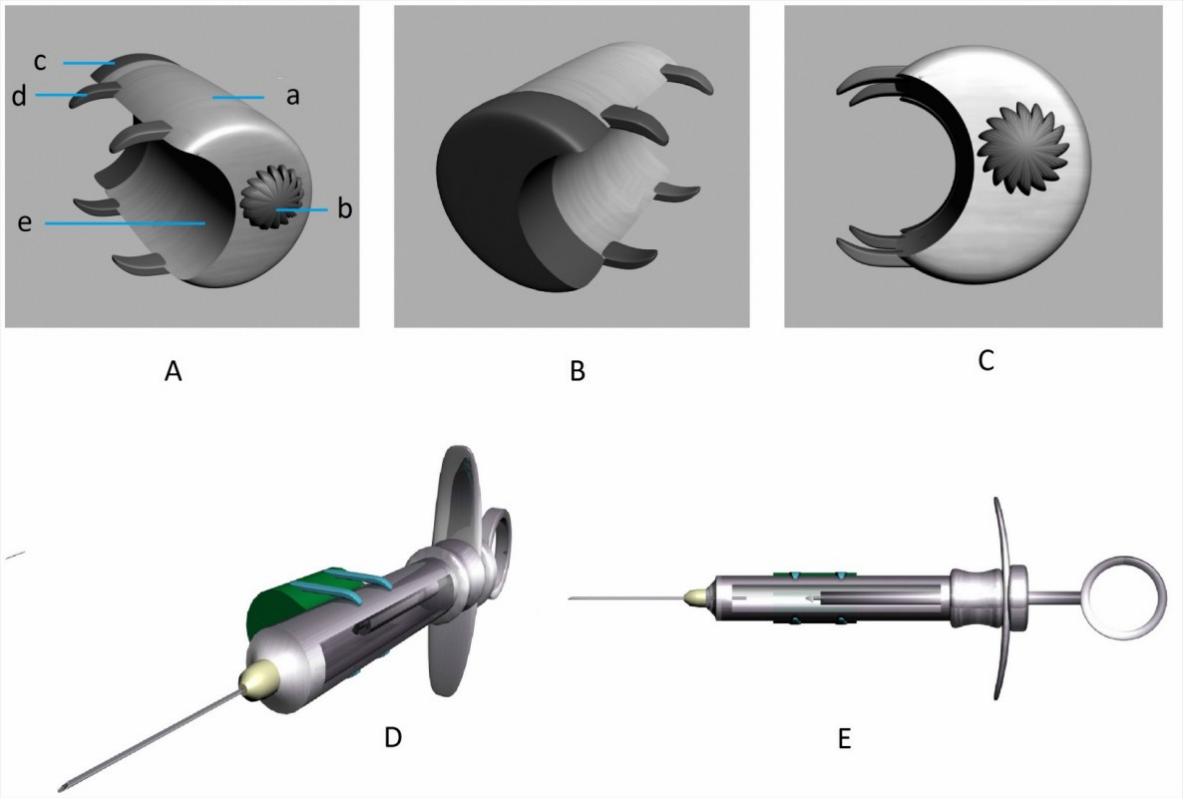
**Structural components of Syringe Micro Vibrator (SMV) and its mounting position on dental syringe barrel**. A) Posterior-anterior view of SMV, structural components consist of: a) stainless steel shell containing motor and eccentrically weighted plate, b) power switch, c) stainless steel cap, d) four flexible attachment arms for firm attachment and e) shell concavity for well adaptation on syringe barrel. B) Anterior-posterior view of SMV and stainless steel cap bearing button cell batteries, C) SMV is applicable to most standard conventional syringes which compensates minute variations of different barrel diameters through four flexible grasping positioning arms and shell concavity, D) SMV mounted on syringe barrel and E) Lateral syringe view indicates that mounting of SMV causes no restriction for the replacement of cartridge.

SMV was designed to provide feasibility to alleviate injection pain and anxiety in clinical practice. Upon mounting on a conventional dental anesthesia injection syringe, its motor is switched on and the clinician then uses normal injection technique to administer the anesthetic. Its parallel mounting on the syringe allows clinician to rotate the syringe while in the mouth, if necessary. Its main structural parts consist of a) stainless steel shell bearing four flexible attachment arms, b) eccentrically weighted plate and motor, and c) two button batteries. SMV is registered as an invention in the field of dentistry and received Iran National Patent number of 63765. Schematic mounting position of SMV on injection syringe is indicated in Figure 1.

## Testing the hypothesis

Micro vibration of injection needle with ultra high frequency and ultra low altitude will alleviate the pain and anxiety during intraoral injections. SMV bears an off-set rotating micro vibration motor and can be easily mounted on any standard dental syringe. Accordingly, "Pain gate" would be shut by stimulating nerves providing reduction in patient pain and discomfort during injection period. SMV makes nerve endings sense micro vibrations at the very early stage, so will remarkably reduce the pain transmission. Implication of SMV would have advantages of: 1) From the aspect of the patient pain management, SMV contributes both physiologically (based on Gate Control Theory of pain) and psychologically (based on the device function as will be explained by dentist to the patient as a modern pain reducing technology), 2) from the aspect of clinician, SMV motor provides vibrations with ultra high frequency to alleviate pain, but since it has ultra low vibration altitude, it has no adverse effect on the clinician dexterity and accuracy during injection and it does not interfere with pin point localization of injection site, 3) it alleviates two types of pain injection including both needle insertion pain and balloon effect due to forceful penetration of anesthetic into the surrounding tissue. The micro vibration slowly reduces such balloon and enhances tissue infiltration of injected anesthesia, 4) it is easy to use and does not provide any inconvenience for the clinician during injection operation due to a- low weight that does not affect the accuracy of clinician, b- small size that keeps well visibility, c- battery powered and lack of wire or hose attachment, 5) firm grasp by four flexible attachment arms it provides: a- convey of efficient vibration to syringe barrel and consequently to needle, b- applicable to all standard conventional syringes and compensates minute variations of different barrel diameters, c- no screw or spare appliance needed for its mounting to or removal from the syringe barrel, so its application is fast and easy, d- it can be attached anywhere along the syringe barrel while it does not cover or mask the cartridge, 6) no need to replace the existing syringes or purchase further spare parts, 7) it has a detachable motor, a stainless steel shell and four attachment arms which are autoclavable and are the only parts in contact with patient during injection. The motor should not be heat sterilized but for disinfestations, it can be easily removed from its shell socket before autoclaving or can be wiped off with a surface disinfestant and 8) use of SMV would save time since it eliminates the period needed for application and onset of topical anesthetic. It also shortens period of anesthesia onset with increase of anesthetic diffusion pace to surrounding tissues since the ultra vibration enhances tissue infiltration of injected anesthetic.

## Simplicity of application

Implication of SMV requires no topical anesthetic or special technique. SMV would weigh approximately 30-50 grams and would be easily mounted on dental injection syringes. The SMV package would bear motor assembly, autoclavable stainless steel shell bearing flexible attachment arms, two button batteries, a recharging unit and power cord. SMV will bring more comfort for both patient and the physician during the process of injection. This device by creating micro vibration would be effective in reducing the pain and anxiety confronted with most types of intraoral injections as palatal, mandibular block, intraligamental and local infiltration.

## Discussion

Most patients feel physically and psychologically uncomfortable about penetration of injecting needle into their oral tissues. Some of them do not convey this feeling to the clinician, so the number of patients fearful of the dental pain experience and feeling discomfort is more than what is seen in clinic, however, in many patients fear of injection contributes significantly to postponement of dental treatment however, less painful injection experience with SMV lowers the future fear of injection in such patients. The "Gate-control" theory [29,30] reveals that using appropriate pressure or vibration can close the neural "gate" so that the central perception of itch and pain is reduced. In other words, the "Pain gate" can be shut by stimulating nerves responsible for carrying the signals which enable the relief of pain through massage techniques, rubbing, pressure, ice packs, acupuncture, electrical analgesia and the application of vibration. Examples of less pain experiences based on Gate theory may be seen in: a- Incision in bare hands while playing in snow feels less pain than normal, b- use of simultaneous cold and vibration in ladies epilating devices as accessory stimuli reduces transmission of main stimulus by local nerve endings in hair removal leading to reduced prick and c- some dentists have developed pain reducing techniques like shaking the syringe in their grasp while dispensing the anesthetic [25].

## Comparative study

A similar device reported in the literature, VibraJect^®^, has controversial performance. Blair [32] recommended the use of VibraJect^® ^for painless injection. In contrast, Yoshikawa *et al *[33] found no significant pain reduction when VibraJect^® ^was applied with a conventional dental syringe. Saijo *et al *[34] evaluated the effectiveness of VibraJect^® ^in combination with an electrical injection device. Injections were given into the alveolar mucosa adjacent to the root apex of the maxillary lateral incisor in 10 volunteers. VibraJect^® ^was randomly applied to either the left or right side of the injection. They found no statistically supports use of VibraJect^® ^but expressed that it offers a simple and easy-to-use solution that can anesthetize patients quickly in a more comfortable manner. They also point out that VibraJect^® ^enables a less painful palatal injection because it delivers small amounts of anesthetic solution over a period of time. Another supporting result was statistically performed by Purray *et al *[35] at Queens University with conclusion that the vibrating syringe attachment resulted in reduced pain levels on receiving intraoral injections. The study performed on 400 patients and showed that VibraJect^® ^statistically reduced the amount of pain score from 4.6 to 1.7 which has never been statistically achieved before. Accordingly it is conclusive that VibraJect^® ^effectiveness is approved, however as indicated in Table 1 SMV has improvements in several structural features including vibration mode bringing enhanced effectiveness to the method of using vibration in dental anesthesia injection. In the table, technical features, performance and a brief summary for specification differences between SMV and VibraJect^® ^are indicated correspondingly. At last, it should be pointed out that SMV is not only a useful accessory device for ordinary patients, but also more useful for pediatric patients and those who have a phobia of intraoral injection or pain. The author believes SMV will bring more comfort for both patient and the physician during the process of injection.

**Table 1:** Comparative study indicating specification differences between Syringe Micro Vibrator (SMV) and VibraJect®

Specifications	**VibraJect**^®^	SMV	Advantage of SMV
Attachment interface	Clip	Four flexible grasping positioning arms and concave shell contact surface	Firm grasping, efficient vibration conveyance, causes no restriction for the replacement of cartridge, applicable to all standard conventional syringes which compensates minute variations of different barrel diameters
Mounting angle on syringe barrel	Angular	Parallel	Most efficient contact, least clinician vision masking, least patient discomfort, allowing the clinician to rotate the syringe while in the mouth, if necessary
Vibration device	Eccentrically weighted shaft	Eccentrically weighted plate	Yield of uniform micro vibration, more precise localization of injection point
Vibration mode	High frequency	Ultra high frequency and ultra low altitude	Efficiently enhance patient pain reduction, and ease of clinician maneuver and accuracy during injection

Accordingly, what expressed in this article about using SMV is based upon potential usefulness of vibratory stimulation for pain since vibration would increase the threshold for dental injection pain as indicated in earlier findings of other researchers [18,23,36]. The author expresses that although SMV is designed to alleviate the injection pain during anesthesia injections, there may be no significant difference in pain perception in patients with a higher pain tolerance. Since pain tolerance cannot be estimated ahead of injection [37], it is recommended to use SMV for most patients.

## Implications of the hypothesis

SMV is a useful accessory device adaptable for dental injection syringe and conventional intra muscular injections to alleviate pain and stress of injection.

## Abbreviations

SMV: Syringe Micro Vibrator.

## Competing interests

The authors declares that he has no competing interests.

## Authors' contributions

AHSB carried out the entire design of the study and draft the manuscript. He read and approved the final manuscript.
